# Immune Response of *Galleria mellonella* against Human Fungal Pathogens

**DOI:** 10.3390/jof5010003

**Published:** 2018-12-26

**Authors:** Nuria Trevijano-Contador, Oscar Zaragoza

**Affiliations:** 1 Albert Einstein College of Medicine, Bronx 10461, NY, USA; 2 Mycology Reference Laboratory, National Centre for Microbiology, Instituto de Salud Carlos III, Majadahonda, 28220 Madrid, Spain; ozaragoza@isciii.es

**Keywords:** *Galleria mellonella*, fungal pathogens, innate immunity, cellular response, humoral response

## Abstract

In many aspects, the immune response against pathogens in insects is similar to the innate immunity in mammals. This has caused a strong interest in the scientific community for the use of this model in research of host–pathogen interactions. In recent years, the use of *Galleria mellonella* larvae, an insect belonging to the *Lepidoptera* order, has emerged as an excellent model to study the virulence of human pathogens. It is a model that offers many advantages; for example, it is easy to handle and establish in every laboratory, the larvae have a low cost, and they tolerate a wide range of temperatures, including human temperature 37 °C. The immune response of *G. mellonella* is innate and is divided into a cellular component (hemocytes) and humoral component (antimicrobial peptides, lytic enzymes, and peptides and melanin) that work together against different intruders. It has been shown that the immune response of this insect has a great specificity and has the ability to distinguish between different classes of microorganisms. In this review, we delve into the different components of the innate immune response of *Galleria mellonella*, and how these components manifest in the infection of fungal pathogens including *Candida albicans*, *Aspergillus fumigatus*, *Cryptococcus neoformans*, and *Histoplasma capsulatum.*

## 1. Introduction

The investigation of infectious diseases has been one of the priority fields in medicine in recent years. The use of animal models is crucial in this area, as it allows us to define the causative agents of these diseases, examine mechanisms of interaction between host/pathogen, and evaluate the efficacy of drugs among other uses. One of the most commonly used models for studying microbial infections involves the experimentation with mice. This model is very versatile because it allows the use of genetically modified animals and the characterization of the role of the host immune response during infection. Despite its importance, the use of mouse models has several ethical and logistical issues associated. The facilities and maintenance of these animals is expensive. In addition, many models of infection can induce acute sepsis and physiological alterations in animals, which can create great discomfort and pain. For this reason, animal experimentation is nowadays regulated by authorities and bioethical committees. To reduce these bioethical problems, there is a strong trend to apply the “3 Rs” rule in experiments that involve animal use, which are: *reduce* the numbers of animals; *refine* the protocols to increase animal comfort and reduce pain; *replace* animals for other models that do not have bioethical problems associated [1]. In addition, the use of mice for the study of some infectious diseases might present other limitations. For example, in the case of fungal pathogens, the development of murine infection models is not straightforward. Except a few cases (such as *Candida albicans* or *Cryptococcus neoformans*), establishment of an infection and disease in mice requires the use of immunosuppressants or may take a considerable amount of time. Therefore, virulence is often assessed by measuring the fungal burden in the animals rather than death. For these reasons, particularly in the field of fungal pathogens, microbiologists have sought “non-conventional” hosts to investigate microbial pathogenesis and for testing antimicrobial drugs [1]. Among them, nematodes (mainly *Caenorhabditis elegans*) and insects are being more widely used to study infectious diseases.

The Insecta class (insects) are a large and very diverse group of invertebrate animals that contains more than one million described species [2]. Among the main orders of insects are the Diptera, Hemiptera, Coleoptera, Hymenoptera, and Lepidoptera, to which *Galleria mellonella* belongs. In nature, they are exposed to a wide variety of pathogens, which has led to the development of protective systems that include resistant physical barriers, such as the cuticle. In addition, they have developed complex and effective immune defense systems.

During its life cycle, *G. mellonella* can be found in the larval, pupal, or moth form and it is known as honeycomb moth. The life cycle of this organism comprises a larva stage (size around 1–3 cm) that transforms into pupae and finally into moth (3–4 cm) [3,4]. The size of the larvae makes their manipulation easy. *Galleria mellonella* has been used prominently as a model to investigate microbial pathogenesis. The application of this model to study the role of virulence factors in disease and efficacy of antimicrobial compounds has been demonstrated in more than 1000 publications on PubMed [5]. For this purpose, mainly adult larvae are used, before they transform into the pupa stage of the life cycle. The structure of this insect is very simple, it consists of a digestive tube, a neural tube that runs from the frontal side through the dorsal part, and a fat body. All of these organs are surrounded by hemolymph.

Given that some aspects of the *G. mellonella* innate immunity are similar to the immunity of mammals, it makes this model a feasible option to study human infections [6]. Recently, the use of this model in the mycology field has been applied to *Aspergillus fumigatus* [7], *Candida albicans* [8,9,10], and *Cryptococcus neoformans* [11].

## 2. Advantages and Disadvantages of the *G. mellonella* Model

This model offers several advantages which can be highlighted: it is an easy model to implement in the laboratory without the need to invest a large amount of money; due to their size and life cycle, the larvae are easy to handle and there is no need for personnel with specialized expertise; it is easy to determine the viability of the larvae; they are affordable for all laboratories; it is possible to administer exact doses of drugs and pathogens; they can be incubated and survive in a very wide range of temperatures (including 37 °C, which is the human body temperature) even though the optimum temperature of growth is 25 °C [12].

Although it is a model widely used in research, there are also some limitations. Although its genome has been recently sequenced [13], it is not possible to perform genetic manipulations. In addition, it can be difficult to contact a reliable company that provides the larvae in optimal conditions. In many cases, these companies do not control the absence of infection prior to shipment. There are also some distributors who grow the larvae specifically for research, but the price to purchase is higher. Although working with *G. mellonella* does not require special equipment, it is recommended to keep the larvae in a confined space to prevent the release of moths. Finally, it requires proper prior planning to have an appropriate supply of larvae of the same size and age for experimentation.

*Galleria mellonella* is commonly used to test virulence of microbial pathogens and for evaluating in vivo activity of antimicrobial drugs. It is also a model used to study in vivo toxicity of food preservatives and antimicrobial drugs [14,15,16].

## 3. Immune Response of *G. mellonella*

The components that comprise the *G. mellonella* immune system are: fatty body, lymph nodes, and the hemocytes that originate from the mesoderm. These insects have an open circulatory system referred to as the hemolymph, which occupies the general cavity of the body (hemocele). The immune response of *G. mellonella* is innate, and it is composed of three parts: physical as well as chemical barriers that protect the insect from the external environment, the humoral response, and the cellular response [17]. The cellular response is mediated by hemocytes (blood cells of *G. mellonella*). The functions of these hemocytes include storage of various types of substances, as well as providing defense of the organism, based on coagulation mechanisms, phagocytosis, nodulation, and melanization. The humoral response is based on different types of soluble molecules secreted against pathogens. This response mainly comprises lytic enzymes (lysozyme), antimicrobial peptides, and melanin.

## 4. Cellular Immune Response

The main component of the cellular immune response is composed of hemocytes, which are cells with phagocytic capacity that have the ability to produce antimicrobial compounds. Hemocytes are involved in all the mechanisms of insect defense. These cells are not responsible for the transport of gases, since there is a complex network of tubes (tracheal trunks, tracheas, air sacs) that fulfill this function [18]. The main processes of cellular response include coagulation, nodulation, phagocytosis, encapsulation, and melanization [19]. Up to six classes of hemocytes have been described in this insect. These include prohemocytes, plasmatocytes, granular cells, coagulocytes, spherulocytes, and enocytoids [5].

Prohemocytes are a small cell type that have a large nucleus and the ability to differentiate into different cell types [20]. Plasmocytes are larger than prohemocytes with an oval shape and are the most common type of hemocytes. Granular cells have a small nucleus and many granules in the cytoplasm. Spherocytes can be found with different shapes and have inclusions of rounded shape. These hemocytes can transport cuticular components. Coagulocytes are hemocytes whose main function is hemolymph clotting, which is an important process because it is the first defense induced by these insects after microbial exposure. Oenocytoids, which represent 5–10% of the total hemocytes, contain cytoplasmic phenoloxidase and participate in the melanization of the hemolymph [21]. Oenocytoids can also secrete nucleic acids that have been described as “a new alarm signal” in the defense of these insects. Although many studies mention that only plasmatocytes are involved in phagocytosis, it has been shown that both plasmatocytes and granular cells participate in this process as well as in encapsulation [5,19,22].

The process of nodulation occurs when pathogens are recognized and killed by trapping them in hemocyte aggregates (nodules), whereas melanization occurs inside the nodules [16,22]. Encapsulation is performed by granular cells and plasmatocytes. Granular cells bind to the microorganism and trigger the union of plasmatocytes that surround the pathogen, forming a multicellular capsule where the pathogen is killed by the release of molecules by both types of cells. 

The process of phagocytosis occurs in two main steps: first, the granular cells make contact with the pathogen, which causes the release of their granular content. This promotes the second step, which is adhesion of plasmatocytes to the pathogen to complete the process [23]. Once internalized, several killing mechanisms are induced, such as the production of superoxide, which leads to the destruction of the pathogen [24,25].

## 5. Humoral Immune Response

The humoral response comprises of many components, such as antimicrobial peptides (AMPs), lytic enzymes, opsonins, as well as molecules with direct activity against pathogens, such as phenoloxidase, which protects the host by catalyzing the formation of melanin.

### 5.1. Antimicrobials Peptides (AMPs)

The humoral immune response of insects is mainly based on the synthesis of antimicrobial peptides (AMPs). The contact with microorganisms induces the transcription of AMP-encoding genes. These molecules play a fundamental role in host defense and are produced mainly in the fat body, in both the digestive and reproductive tract, and subsequently are released into the hemolymph [26]. In some models, the induction of AMPs is transient, being very strong within the first 6 hours of infection but diminished after 3 days. AMPs are divided into cationic and anionic antimicrobial peptides. Cationic AMPs are basically classified into three groups based on their structure: (a) linear α-helical peptides without cysteine residues (among them, cepropins and moricins are active against bacteria and filamentous fungi [27,28,29]); (b) peptides with a structure stabilized with disulfide bridges, such as the cysteine-rich peptides (for example, gallerimycin and galiomycin, which are defensive peptides against fungi) [5,29,30]; (c) peptides with proline and/or glycine residues, such as Gm proline-rich peptide 1, with the ability to inhibit growth against yeast [31] and glycin-rich AMPs, such as gloverin, which inhibits the synthesis of membrane proteins in bacteria [5,31,32,33]. The anionic defense AMPs are activated against pathogens resistant to cationic peptides. In *G. mellonella*, two of these peptides, purified from the hemolymph, AP1 and AP2, have been described [31,34].

### 5.2. Lytic Enzymes (Lysozymes)

Lysozymes are an important element within the humoral response against pathogens found in the hemolymph of insects belonging to the orders Lepidoptera, Diptera, Coleoptera, and Hymenoptera [35,36,37,38]). Lysozyme is an antimicrobial enzyme that damages the bacterial cells by hydrolyzing β-1,4 linkages. Lysozyme also presents antifungal activity through enzymatic activity of the fungal cell walls, which results in growth inhibition [34,39,40,41].

### 5.3. Melanization

Melanin is a compound that is synthesized in response to foreign particles and plays a crucial role in sclerotization, wound healing, and in defense reactions. Melanin produces the accumulation of nodules, which can be visible in histological sections whose main function is to contain the replication of microorganisms [42,43]. The enzyme that catalyzes the synthesis of melanin is a phenoloxidase (PO) that oxidizes phenols to quinones, which subsequently polymerize non-enzymatically to melanin. Phenoloxidase is found in the hemolymph and hemocytes in its inactive form prophenoloxidase (PPO). PPO is activated by cell wall components of fungi and bacteria (lipopolysaccharides, peptidoglycans, and β-1,3-glucans) [44,45,46].

### 5.4. Opsonins

*Galleria mellonella* produces hemolymph proteins that act as opsonins. These proteins recognize components in the cell wall of different microorganisms, such as bacteria and fungi. Most of these molecules recognize and bind to lipopolysaccharides (LPS), peptidoglycan, and β-1,3-glucan [47,48,49,50]. apolipophorin-III (apoLp-III) is a protein that facilities lipid transport [51] and it has been associated with pathogen recognition and apoptosis [52]. Peptidoglycan recognition proteins (PGRPs) bind to bacterial peptidoglycan of the cell wall, causing the hydrolysis of the pathogens [5]. Hemolin is a member of the immunoglobulin superfamily. There are studies that have observed an increase in the production of hemolin after infection with bacteria and viruses [53,54].

## 6. *Galleria mellonella* as a Model to Study Fungal Pathogens

Invasive fungal diseases have become a major life threat to a large population of patients, mainly those that are immunosuppressed. Besides a few human fungal pathogens (such as *C. albicans* or *C. neoformans*), most of the fungal species present low virulence in mice. Therefore, the use of alternative models, and in particular, *G. mellonella*, has offered an alternative means in which to investigate the pathogenic mechanisms of most of the pathogenic fungal species. Recent studies have revealed that the innate immune response has great specificity, in addition to having the ability to distinguish between different classes of microorganisms [55]. *Galleria mellonella* was first described as a model for studying human fungal pathogen in the yeast *C. albicans* [9]. It is currently a model used to assess virulence of fungi such as *A. fumigatus*, *Paracoccidioides lutzii*, *Histoplasma capsulatum*, and *C. neoformans* [6,11,56,57,58]. In this last case, it has been even used to assess the virulence of a large collection of mutants [59]. *Galleria mellonella* model can also be used to assess toxicity and efficacy of antifungal agents during infection. The results obtained with this model have been shown to have a strong correlation with mammal models [60,61].

## 7. Response of *G. mellonella* to Different Fungi

### 7.1. Candida spp.

*Candida spp.* are the most common opportunistic fungal pathogen of humans, which can transform from superficial mucosal infections to systemic infections [62,63,64]. Bloodstream infections (BSIs) caused by *Candida spp.* remain a frequent cause of morbidity and mortality, particularly within the immunocompromised population [65,66,67]. The virulence of several *Candida spp.* (*C. albicans, C. glabrata*, *C. tropicalis C. krusei*, *C. haemulonii*, and *C. auris*) has been evaluated in *G. mellonella* [68,69,70,71,72,73]. 

The most common pathogen is *C. albicans*, and multiple studies have shown that this yeast can effectively kill *G. mellonella*. Both the cellular and humoral responses are important to control *Candida* infection. Different conditions that activate these responses protect against the infection by *C. albicans*. For example, physical stress, pre-incubation of the larvae at high (37 °C) or low (4 °C) temperature, or preexposure to non-lethal doses of *C. albicans* are conditions that increase the concentration of hemocytes as well as the expression of AMPs encoding genes. This has been shown to protect against later challenge with *C. albicans* [74,75,76]. Interestingly, some antifungal compounds that protect during *Candida* infection (such as caspofungin and micafungin) also have immunomodulatory effects and “boost” the immune response of *G. mellonella* during infection [14,74]. 

Inhibition of hemocyte function with cytochalasin and nocodazole enhances the susceptibility of the larvae to the infection [75]. After challenge with *C. albicans*, *G. mellonella* elicits activation of both the cellular and humoral responses. It has been shown that the number of circulating hemocytes fluctuate during the first hours of infection [76] but tend to decrease with time, in association with the severity of the disease [73]. Hemocytes induce nodulation at the site of *C. albicans* infection to avoid fungal replication. This nodulation is accompanied by the early accumulation of melanin at these sites [77].

Recognition of *C. albicans* by *G. mellonella* cells depends on several receptors and proteins. Base on current knowledge, one of the main receptors involved in this recognition is GmCD8 [77], which can also recognize other microbial pathogens. This receptor is not required for the formation of nodules by hemocytes and seems to act as an opsonin that directly induces phagocytosis of the fungal cells. Another protein that binds to the *C. albicans* surface is apolipophorin III [78], which is an apolipoprotein involved in lipid trafficking during insect flight [79]. Apolipophorin can exist in lipid-free or lipid-bound forms, being active when bound to lipophorin particles. In the case of *C. albicans*, apolipophorin III can bind to the surface of this pathogen and trigger the transition from yeast to hyphae, suggesting that this *C. albicans* can use some elements from the *G. mellonella* immune system to activate protective and adaptive responses. Furthermore, infection with *C. albicans* can alter the proportion of apoLp-III-free and apoLp-III-bound in the hemolymph [80], suggesting that they also participate in the recognition of fungal pathogens. 

The main epitopes that are recognized by immune cells are located at the cell wall. In particular, β-glucans are the polysaccharides that elicit some of the strongest responses in mammalian cells [81]. In the case of *G. mellonella*, β-glucans can also activate some of the immune responses. Administration of β-glucan augments the density of hemocytes in the hemolymph and also increases the expression of some AMP-encoding genes [82]. This effect is in agreement with the finding that administration of β-glucan or laminarin can protect the larvae against lethal doses of *C. albicans* cells [83]. 

Elements of the humoral response are also involved in the defense against *C. albicans*. In particular, lysozymes can kill *C. albicans* [34]. Interestingly, this fungicidal effect is not directly related to degradation of the cell wall, but to induction of apoptosis in the yeast cells [84]. Another peptide that has been involved in the immune response against this pathogen is the anionic peptide 2 (AP2). However, this peptide does not kill the yeast cells, but has a fungistatic effect and, furthermore, enhances the killing activity of lysozyme [34].

### 7.2. Aspergillus fumigatus

*Aspergillus fumigatus* is the most common mold involved in human infections and the most isolated of the *Aspergillus* [56]. *A. fumigatus* produces allergic, chronic, and invasive disease depending upon the host immune system [85]. The *G. mellonella* model has been widely used in this pathogen for studies of virulence, survival, susceptibility, and antifungal resistance [56,57]. Killing of larvae by *A. fumigatus* is associated with massive replication of the fungus and filamentation. Furthermore, some toxins produced by *A. fumigatus*, such as fumagillin, can decrease the phagocytic and antifungal activity of hemocytes, which highlights another mechanism by which this mold can interfere with the larva immune response [86]. 

Infection of *G. mellonella* with high *A. fumigatus* doses results in dissemination of the fungus through the larva body after 24 h. However, *G. mellonella* induces early immune responses. For example, after 2 h of inoculation, there is an increase in the hemocyte density in the hemolymph [85]. Furthermore, fungal cells are contained in melanized nodules, as demonstrated with *Candida* spp. Proteomic analysis also indicate that *A. fumigatus* induces the accumulation of AMPs and immune receptors [85]. 

Interestingly, infection with non-lethal doses is enough to prime the larva immune response by inducing the expression of AMPs and the accumulation of hemocytes. This response is sufficient to protect against infection with higher and lethal doses of the fungus [87]. However, it has also been indicated that an exaggerated immune response might have negative effects for *G. mellonella*. In contrast to the situation in mice, *A. fumigatus* mutants that do not produce melanin have enhanced virulence in *G. mellonella* [88]. This phenotype is not due to an increased germination of the conidia. In contrast, *A. fumigatus* pigmentation mutants induce stronger darkening of the larvae after inoculation, suggesting that they overactivate the immune response of the insect. Furthermore, coinfection with wild type and pigmentation mutants results in a trend to increased virulence. Jackson et al. suggested that absence of melanin at the surface unmasks some fungal PAMPs (such as β-1,3-glucan), which triggers an excessive immune response that has deleterious effects on the larvae [88].

### 7.3. Cryptococcus neoformans

*Cryptococcus neoformans* is a basidiomycetes yeast widely distributed in the environment that can behave as a pathogen in susceptible patients [89,90]. *Cryptococcus neoformans* infects many people, yet few develop disease, most commonly cryptococcal meningoencephalitis. The infection produced by this pathogen occurs mainly in HIV patients. *Cryptococcus* has the capacity to adapt to several environmental hosts and evade the immune response through multiple mechanisms [91,92,93,94].

This pathogen has a polysaccharide capsule that plays multiple roles during infection. It protects the fungus against stress factors, but it can also act as a virulence factor and alter the host immune response [95]. Another interesting factor of this yeast is its ability to evade killing by phagocytic cells after phagocytosis, so it is considered a facultative intracellular pathogen [90,96,97,98]. In addition, *Cryptococcus* can also develop some typical morphological transitions that contribute to immune evasion. The most characteristic consist basically of the increase in the size of the capsule [95,99,100] and the formation of titan cells [101,102,103,104,105].

*Cryptococcus neoformans* can infect and kill larvae [11], and this insect has been frequently used to investigate virulence of clinical and environmental strains. The responses of *C. neoformans* in *G. mellonella* are similar to those observed in mice. For example, capsule growth and titan cells have been observed during *G. mellonella* infection [106]. The immune response of *G. mellonella* against this pathogen has been also investigated [6]. Interestingly, infection with *C. neoformans* does not result in early melanization at the infection sites, as it happens with *Candida*. This is most probably due to the presence of the capsule and to the different cell wall composition between these two fungal species. *Galleria mellonella* induces the accumulation of AMPs after cryptococcal challenge, which is activated in part by the presence of the capsule. Furthermore, *C. neoformans* cells are avidly phagocytosed by *G. mellonella* hemocytes and, interestingly, this fungal pathogen can survive and replicate after internalization, indicating that it can also behave as an intracellular fungal pathogen in *G. mellonella* hemocytes [6].

### 7.4. Other Fungal Pathogens

The virulence of other fungal pathogens in *G. mellonella* has been also studied. For example, *Fusarium* spp. (in particular, *F. oxysporum* and *F. solani*) are filamentous fungi that can also cause different diseases in immunocompromised patients. These fungi are widely found in the environment, where they can also behave as frequent plant pathogens. *Fusarium oxysporum* can cause disease in *G. mellonella*, and its virulence depends on the inoculum size and conidia germination and hypha proliferation [107]. Virulence in *G. mellonella* correlates with the pathogenicity in mice [108]. Furthermore, *Fusarium* macroconidia are more virulent than microconidia in *G. mellonella*, despite both forms being effectively phagocytosed by the hemocytes [108]. An elegant proteomic analysis using iTRAQ labeling revealed the changes that *F. oxysporum* induces in protein profile in *G. mellonella*. Munoz-Gomez et al. found that *G. mellonella* response to *F. oxysporum* is highly dynamic and dependent on the inoculum size (10^4^ or 10^6^ conidia per larva) and on the incubation temperature (25 or 37 °C ) [109]. 

**Figure 1:**
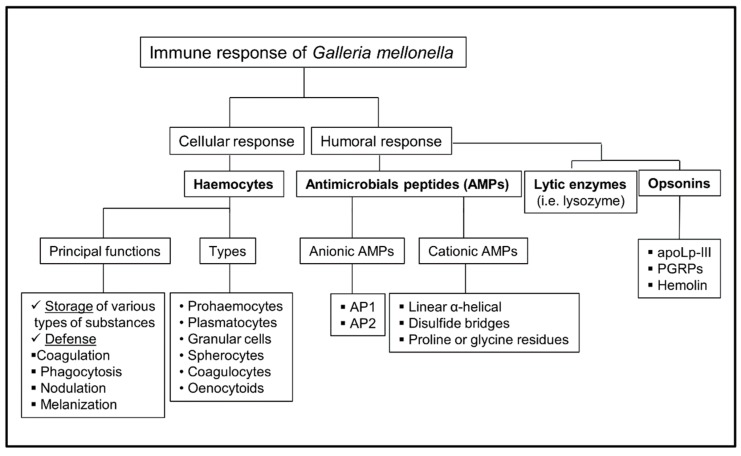
Scheme of the immune response of *Galleria mellonella.* PGRPs: Peptidoglycan recognition proteins; AMPs: antimicrobial peptides.

Dimorphic fungi are clinically considered as those that present a filamentous morphology in the environment but behave as yeasts at 37 °C in the host. These fungi can cause disease in both immunocompromised and immunocompetent individuals. Main dimorphic fungi are *Histoplasma capsulatum*, *Coccidioides immitis*, *Blastomyces dermatitidis*, *Paracoccidioides brasiliensis*, and *P. lutzii*. Infection models of these fungi in mice can be very long (even months, see [110]), which poses a great limitation to assessing their virulence traits. For this reason, the *G. mellonella* model has been used to investigate the virulence of some of these fungi, such as *H. capsulatum* and *P. lutzii* [58,111,112]. Interestingly, the virulence of these two fungi in *G. mellonella* does not correlate with the inoculum size [58]. In contrast, both fungi induce early melanization of the larva in a dose-dependent manner. These authors also compared the virulence of two *H. capsulatum* strains that differ in their cell wall composition. In particular, the strain that lacks a layer of α-glucan on the surface that unmasks the β-glucan layer, has significantly reduced virulence, suggesting that activation of the *G. mellonella* immunity by the β-glucan component elicits a protective response against this pathogen (Figure 1).

## 8. Future Perspectives

Animal experimentation is nowadays subjected to strict laws and regulations. Furthermore, society has increasingly become concerned about the use of animals in research due to the pain derived from the experimental procedures. For these reasons, in the last decades, there has been a great interest to implement and expand the “3Rs” rule. The application of *G. mellonella* in biomedical research is becoming a useful model for investigating microbial pathogenesis. However, it is important to understand the limitations of this model too. For example, since this lepidopteran does not have adaptive immunity, it is not feasible to propose that *G. mellonella* can fully replace the use of other complex models, such as mice. However, the simplicity of the *G. mellonella* model to analyze microbial virulence and the efficacy of antibiotics and antifungals provides an excellent alternative to reduce the number of experimentation animals. The lack of genetic tools in *G. mellonella* poses another limitation to investigate the role of specific elements of the immune system. However, the recent elucidation of its genome will open new perspectives in the experimental approaches carried out with this insect.
